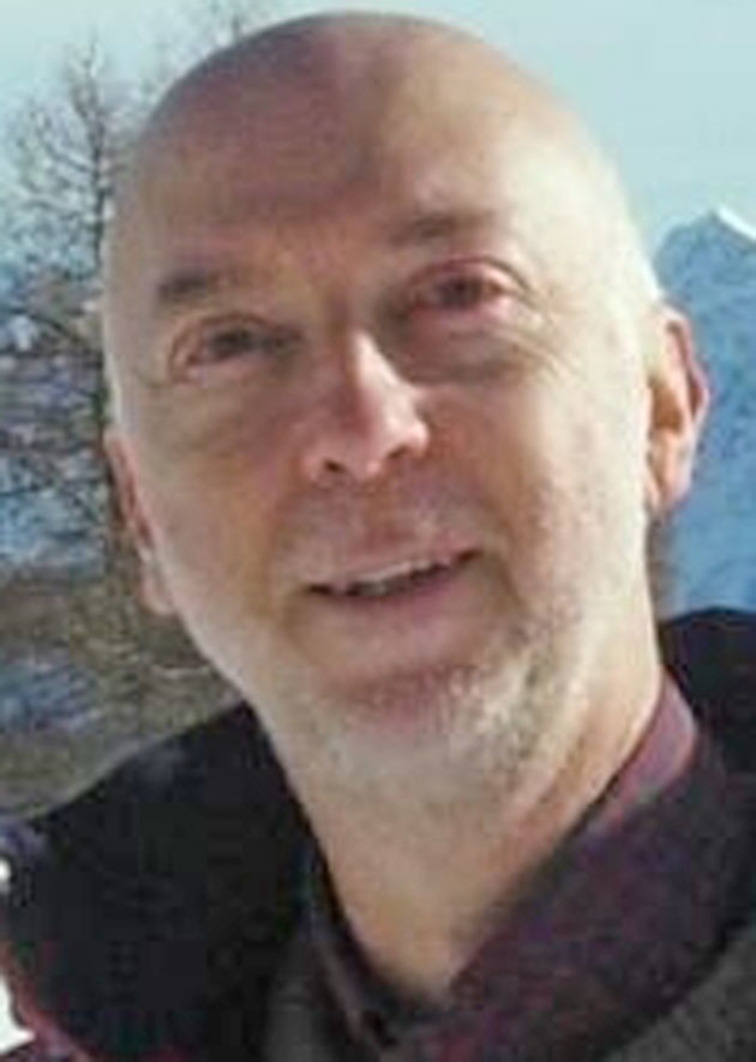# Rational Design of Semiconductor Heterojunctions for Photocatalysis

**DOI:** 10.1002/chem.202101764

**Published:** 2021-08-01

**Authors:** Giovanni Di Liberto, Luis A. Cipriano, Sergio Tosoni, Gianfranco Pacchioni

**Affiliations:** ^1^ Dipartimento di Scienza dei Materiali Università di Milano – Bicocca Via R. Cozzi 55 20125 Milano Italy

**Keywords:** band gaps, band offsets, density functional calculations, heterojunctions, photocatalysis

## Abstract

Electronic structure calculations provide a useful complement to experimental characterization tools in the atomic‐scale design of semiconductor heterojunctions for photocatalysis. The band alignment of the heterojunction is of fundamental importance to achieve an efficient charge carrier separation, so as to reduce electron/hole recombination and improve photoactivity. The accurate prediction of the offsets of valence and conduction bands in the constituent units is thus of key importance but poses several methodological and practical problems. In this Minireview we address some of these problems by considering selected examples of binary and ternary semiconductor heterojunctions and how these are determined at the level of density functional theory (DFT). The atomically precise description of the interface, the consequent charge polarization, the role of quantum confinement, the possibility to use facet engineering to determine a specific band alignment, are among the effects discussed, with particular attention to pros and cons of each one of these aspects. This analysis shows the increasingly important role of accurate electronic structure calculations to drive the design and the preparation of new interfaces with desired properties.

## Graphical abstract

**The nature, structure** and electronic properties of some example of binary and ternary heterostructure relevant in photocatalysis experiments is reported in this work, focusing on important computational aspects such as the band gap problem, quantum size effects, band edges alignment, surface termination and on the insights that simulation can provide when designing new materials.

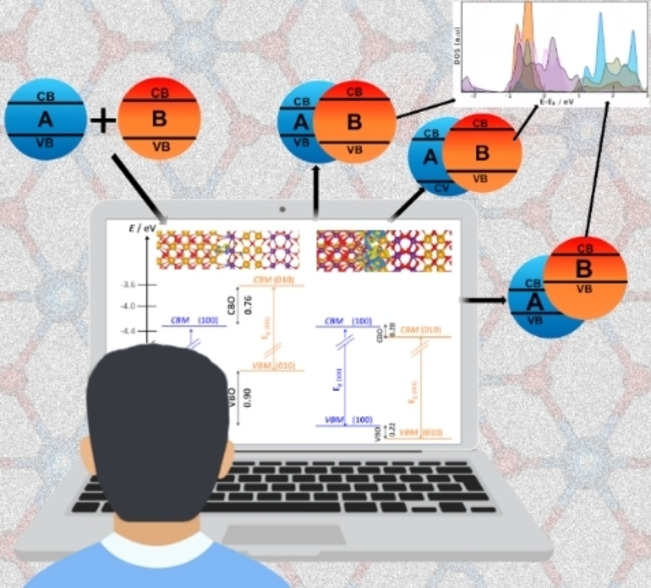

## 1 Introduction

A variety of strategies have been used to increase the efficiency of photocatalytic materials, via doping, nano‐structuring, formation of a semiconductor heterojunction, use of co‐catalysts, or a combination of these approaches.[^1^, ^2^, ^3^, ^4^, ^5^, ^6^, ^7^, ^8^, ^9^, ^10^] The applications of heterojunctions in photocatalysis cover various aspects of modern life.[^11^, ^12^, ^13^, ^14^, ^15^, ^16^, ^17^, ^18^, ^19^] The key requirements for the design of an efficient photocatalyst are (1) a good visible light absorption, (2) a favorable position of the edges of the valence and conduction bands (VB and CB) with respect to target redox species, (3) an efficient charge carriers separation upon excitation, (4) a high mobility and low recombination rate of the charge carriers, and last but not least (5) a good chemical stability.[^20^, ^21^, ^22^, ^23^, ^24^, ^25^, ^26^, ^27^] Composite materials in which two or more units are in intimate contact thanks to the formation of a junction region can in principle successfully address all these aspects.[^2^, ^3^]

**Figure 1:**
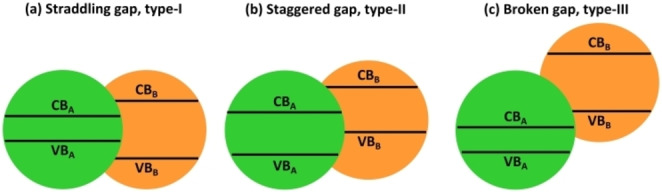
Three types of semiconductor heterojunctions: (a) straddling gap, Type‐I (b) staggered gap, Type‐II (c) broken gap, Type‐III.

One can identify three types of heterojunctions depending on the alignment of the VB and CB edges of the interface: straddling gap (Type‐I), staggered gap (Type‐II) or broken gap (Type‐III), Figure 1.

When the band edges are positioned according to a Type‐II alignment, then the photogenerated electrons and holes migrate towards different components of the heterojunction, hindering recombination and enhancing the efficiency of the redox processes. Sometimes the advantage of creating a heterojunction consists in the possibility to protect a chemically unstable low‐band gap semiconductor by coating it with a corrosion resistant thin oxide film;[^28^, ^29^, ^30^, ^31^] this can lead to a visible‐light active and chemically stable photocatalyst.

The possibility to engineer and design interfaces between different semiconductors opens in principle a variety of systems to investigate. In doing this, one has to take into account a number of parameters, such as the choice of the surface terminations that are interfaced, the nature of the chemical bonds, the occurrence of a charge transfer at the interface, the possibility to growth nanofilms exploiting quantum confinement effects, etc. An important role in this context is played by electronic structure calculations based on Density Functional Theory (DFT). Using advanced DFT methods we studied a series of semiconductor heterojunctions and identified some important trends. In this Minireview the systems that will be discussed have been selected based on their relevance for practical applications, on the existence of experimental results showing the possibility to construct the junctions, and in order to identify specific effects that determine the final behavior of the junction. This gives us the possibility to discuss the underlying physical principles behind semiconductor heterojunctions for photocatalysis, and to show how is it possible to conceive new atomic‐scale interfaces and predict their performances.

## 2 Methodological Aspects

To address semiconductor heterojunctions with DFT calculations one has to deal with various methodological issues. Some are of very general nature, such as i) the problem of the determination of the band gap within the DFT formalism,^[32]^ ii) the role of quantum confinement when nanostructured materials are considered.^[33]^ Other problems are more specific to heterojunctions modelling, such as iii) the necessity of properly align the energy levels of two or more solids connected via formation of an interface. These three aspects will be discussed in this section.

Other methodological problems are intrinsically related to the creation of the interface, and involve the role of surface termination, the problem of strain that can result by the use of the same supercell to describe two materials with different lattice constants, etc.^[34]^ Some of these aspects will be discussed below in connection to specific examples.

Most of the systems discussed below have been studied using the CRYSTAL code[^35^, ^36^] which makes use of localized basis functions; for a few cases the VASP periodic code based on plane waves has been used.[^37^, ^38^, ^39^] The use of two computer codes is motivated by the different efficiency in calculating the band structure with hybrid functionals; on conventional CPUs the atom‐centered CRYSTAL code does not undergo relevant loss of efficiency when doing hybrid functional calculations. However, the increased computing power in the new generation of architectures makes feasible hybrid functional calculations also with plane‐wave codes, as VASP. On the contrary no major qualitative differences are found among the various hybrid functionals tested. Further details can be found in the original publications.

### 2.1 The band gap problem

The problem of describing the Kohn‐Sham (KS) band gap in semiconducting materials has been widely discussed in the past.^[32]^ There is general consensus that hybrid functionals, where a portion of exact exchange is added to the DFT exchange functional, offer a better description of this important property.[^40^, ^41^, ^42^, ^43^] Therefore, all the results discussed in this Minireview are based on hybrid functional DFT calculations. However, several kinds of hybrid functionals have been proposed over the years. They usually differ by the choice of the GGA functional^[44]^ approximating the semi‐local part, and by the amount of exact exchange added to the DFT functional. Usually this is fixed and is not material dependent. However, an alternative is represented by the dielectric‐dependent (DD) functionals where the amount of exact exchange is obtained self‐consistently from the dielectric constant of the material.[^45^, ^46^, ^47^, ^48^, ^49^, ^50^] In a recent study we compared the performances of various popular hybrid functionals, including standard (PBE0, B3LYP, HSE06, etc.) and dielectric‐dependent (DD) functionals.^[42]^ The conclusion is that there is no general improvement in the use of DD functionals compared to PBE0, HSE06 and B3LYP, although some systems are better described at the DD level. Thus, standard hybrid functionals (PBE0 or HSE06) are recommended for non‐magnetic bulk 3D metal oxides, while layered materials benefits from the use of DD, range‐separated functionals.

A specific problem related to the study of heterojunctions where two or more materials are interfaced, is that a single functional must be used for the whole system, thus limiting the use of material dependent functionals such as the DD ones. An important requirement is thus that the functional used provides a balanced description of both components.

### 2.2 Band offsets

To describe the band alignment in a semiconductor heterojunction one can follow three different strategies. The first one consists in using an alternating sequence of slabs A/B/A/B/A/B… repeated along the non‐periodic direction of the supercell (“alternating slabs junction” approach);[^51^, ^52^, ^53^] in this model an interface between A and B is defined and optimized, but the slabs are not terminated by a surface exposed to vacuum. This has the advantage that there is no need to model the surface of a material (provided that no important surface effects are present). A similar approach makes use of a sequence of surface terminated slabs separated by a vacuum region (…), A/B/…/A/B/…/A/B/… . In this “surface terminated junction” approach, two surfaces are present, one for A and one for B, and, as in the previous model, the A/B interface is explicitly taken into account.^[54]^ The third approach is also the simplest one and is based on the calculation of the properties of the separated, non‐interacting, A and B components of the junction (“independent units”). These can be calculated using bulk unit cells,[^55^, ^56^] or finite slab models reasonably converged with respect to the thickness.[^55^, ^56^, ^57^]

Once the model has been defined, the next step consists in calculating the band edges and offsets (VBO and CBO) of the two materials, choosing a proper reference to align them. This can be done following the “electrostatic potential line‐up” method where the plane‐averaged electrostatic potential (V) of the heterostructure and the separated components are calculated.^[51]^ Then, the valence band maximum, VBM, and the conduction band minimum, CBM, of the composing units are aligned using as a common reference the macroscopic average of V or V stationary points.[^51^, ^58^, ^59^, ^60^, ^61^, ^62^] An alternative approach, which has a similar accuracy, is to use the core levels (e. g. the energy of the 1 s orbitals).[^21^, ^63^, ^64^]

### 2.3 Quantum confinement

**Figure 2:**
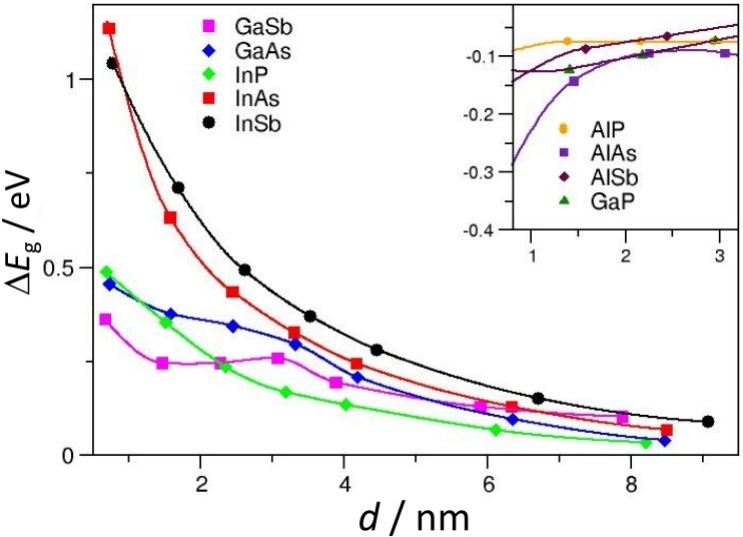
Computed band gap (E_g_^slab^) with respect to the bulk (E_g_^bulk^) for 2D slabs of III−V semiconductors as a function of thickness. Inset: magnified region shows the trend for systems where the gap quickly converges to the bulk value.

When ultrathin films of a semiconductor are grown on top of another material electronic effects due to quantum confinement can emerge. The impact of quantum confinement on the band gap of 2D nanostructures of group III–V semiconductors has been studied by considering nine materials, AlP, AlAs, AlSb, GaP, GaAs, GaSb, InP, InAs, InSb (CRYSTAL code, HSE06 functional).^[65]^ Films from ∼0.5 to ∼9 nm were considered, and the properties gradually converged to the bulk ones, Figure 2.

The variation of the properties with the film thickness provides a measure of the role of quantum confinement.^[33]^ In general, the CB is affected more by the reduced dimensionality while the VB is almost insensitive. InSb and InAs are more sensitive to quantum size effects[^66^, ^67^] and show the largest change of band gap by reducing the film thickness; on the contrary, AlP, AlAs, AlSb and GaP are almost insensitive to nanostructuring. The different behavior of InSb and InAs is due to the different electron‘s and hole‘s effective masses, where the first term is dominant. The electron and hole effective masses of a semiconductor can be computed from the curvatures of the bands corresponding to the CBM and VBM,^[68]^ and provide an indication of the mobility of the charge carriers. The hole‘s effective mass is always higher than the electron‘s one, indicating higher mobility of the latter.

## 3 Type‐II Heterojunctions: Joining Different Semiconductors

In this Section we present five cases of Type‐II heterojunctions as described by DFT calculations. In the first four cases anatase TiO_2_ is interfaced with various semiconductors: halide perovskites,^[69]^ ternary compounds such as BiVO_4_ and SrTiO_3_,[^70^, ^71^] or III–V semiconductors such as InP;^[72]^ in the last example BiVO_4_ is interfaced with WO_3_.^[73]^

### 3.1 TiO_2_/CsPbX_3_

In this first example we consider the role of surface termination. Over the past few years there has been a growing interest towards organic‐inorganic halide perovskites, ABX_3_, where A is an organic cation or an alkali metal cation, B an inorganic cation, and X is the halogen.[^74^, ^75^, ^76^, ^77^, ^78^] These materials find application in solar cells, light emitting diodes, photocatalysis, etc.[^79^, ^80^, ^81^, ^82^, ^83^, ^84^, ^85^] CsPbX_3_ perovskites have been used in combination with oxide films, such as TiO_2_, that protect them from chemical attack[^82^, ^86^, ^87^, ^88^, ^89^, ^90^] and provide a Type‐II alignment of the band edges.[^20^, ^69^, ^77^, ^91^] The most relevant polymorphs of CsPbX_3_ materials are the cubic (α) and orthorhombic (γ) phases.[^76^, ^78^, ^92^] Using the CRYSTAL code and the HSE06 functional the properties of α‐CsPbX_3_ (X=Cl, Br, I) 2D nanostructures have been studied.^[93]^

**Figure 3:**
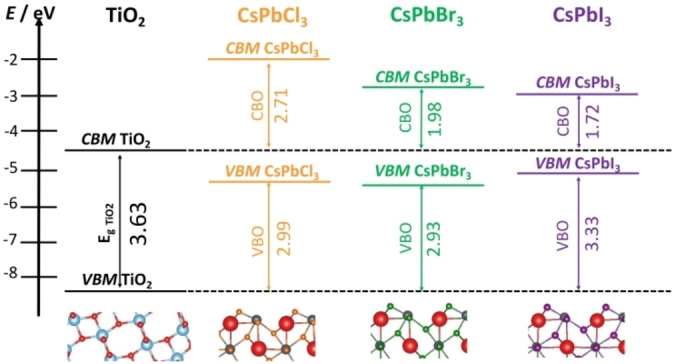
Band edges offset of CsPbX_3_(110) and TiO_2_(101) moieties in CsPbX_3_/TiO_2_ heterojunctions (DFT/HSE06 results).

The (001) surface of α‐CsPbX_3_ (X=Cl, Br, I) 2D nanostructures has two main terminations, PbX_2_‐ and CsX‐, while the (110) one is characterized by three terminations, X, Cs, or PbX. This offers a great variability in designing the junction with TiO_2_. The PbX_2_‐terminated slabs have band edges lower in energy than the CsX‐terminated ones, but their stability is similar. In the case of the (110) surface, X‐termination displays the lowest surface energy. Since the corresponding band edges are in closer agreement with the experiment, it is likely that this is the termination of (110) surfaces in real samples. Taking the most stable terminations, the band edges of the (110) slabs are always below those of the (001) ones, so that it is possible in principle to take advantage of this feature to design systems that induce charge carriers separation. Using the independent units model, without explicitly simulating the interface, a qualitative estimate of the band alignment in CsPbX_3_/TiO_2_ has been obtained, showing a Type‐II alignment for all three perovskites, Figure 3.

This example shows the importance of the selection of the proper facet to construct the heterojunction, a result that has been obtained without the explicit consideration of the interface.

### 3.2 TiO_2_/BiVO_4_

**Figure 4:**
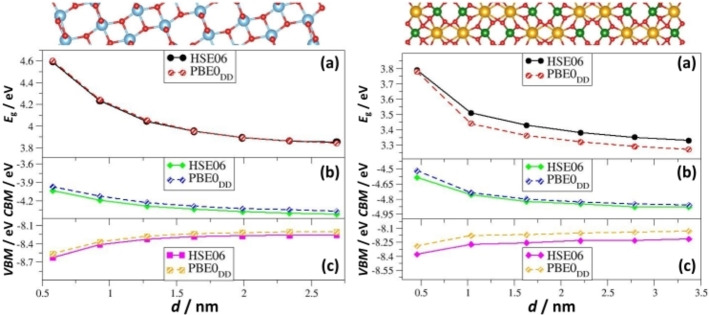
Left: TiO_2_(101); right: BiVO_4_(010). (a) band gap (E_g_), (b) Conduction Band Minimum (CBM), (c) Valence Band Maximum (VBM).

TiO_2_/BiVO_4_ is a highly active system in photocatalytic water splitting.[^94^, ^95^] We use this example to discuss the role of slab thickness and surface termination (CRYSTAL calculations, HSE06 and PBE0_DD_ functionals).^[96]^ Slabs of about 2 nm thickness for both TiO_2_(101) and BiVO_4_(010) components, Figure 4, showed converged structural and electronic properties, with a marginal effect of the choice of the functional. However, using thinner slabs results in considerably shifted VBM and CBM values, Figure 4, showing the importance to check that the results are converged with respect to slab thickness.

**Figure 5:**
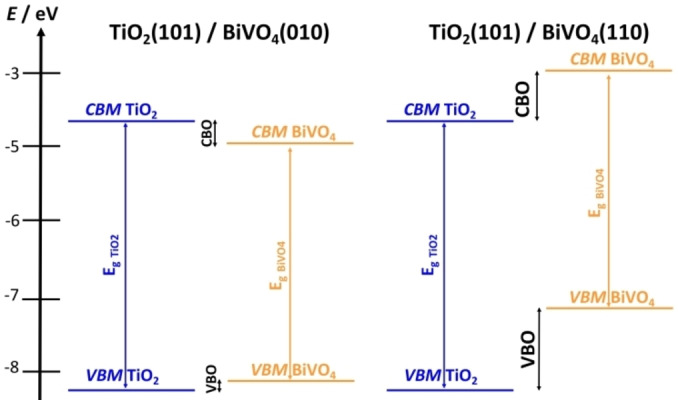
(a) Type‐I band alignment of TiO_2_(101)/BiVO_4_(010); (b) Type‐II band alignment of TiO_2_(101)/BiVO_4_(110) (HSE06 results).

The band edges of the independent units (no explicit interface) were used to analyze the type of alignment in TiO_2_/BiVO_4_ comparing two BiVO_4_ surfaces, (010) and (110). If TiO_2_(101) is interfaced with the BiVO_4_(010) surface the alignment is of Type‐I, and the offset of the VB is negligible, Figure 5, thus reducing the driving force for the separation of the charge carriers. On the contrary, TiO_2_(101)/BiVO_4_(110) is a Type‐II heterojunction, where the band edges of BiVO_4_ are higher in energy that those of TiO_2_. Thus, the TiO_2_(101)/BiVO_4_(110) interface outperforms the TiO_2_(101)/BiVO_4_(010) one showing once more the importance of the surface termination. This prediction, consistent with the experimental observations,^[70]^ is obtained with the simple approach of the “independent units” but is nevertheless useful to assess the nature of the junction and represents a case of facet engineering.

### 3.3 TiO_2_/SrTiO_3_

The interface of anatase TiO_2_(001) with SrTiO_3_(001) is the next example. Differently from the previous two cases, here an explicit interface was constructed (CRYSTAL calculations, PBE0_DD_ functional).^[97]^ TiO_2_/SrTiO_3_ represents an archetype of nearly‐epitaxial matching between the two materials, due to the favorable lattice mismatch between the (001) surfaces of the two semiconductors which minimizes the strain.[^71^, ^98^] However, two surface terminations are possible for SrTiO_3_, SrO and TiO_2_ termination, giving rise to two different interface models.

**Figure 6:**
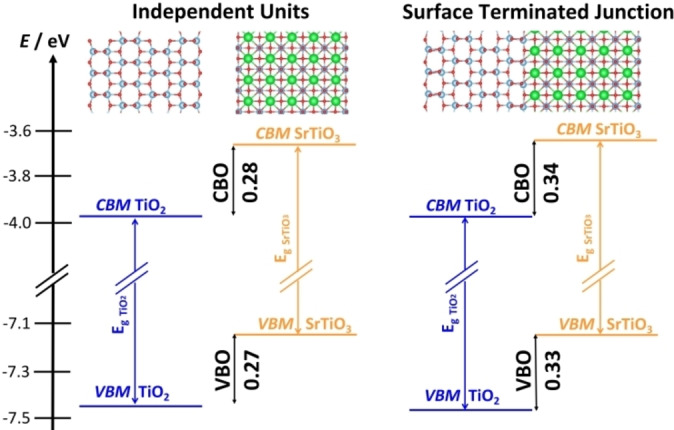
Band alignment for the TiO_2_(001)/SrTiO_3_(001) heterojunction, where SrTiO_3_ is terminated with a TiO_2_ layer. (a) independent units; (b) surface terminated junction models (explicit interface). Blue: TiO_2_; orange: SrTiO_3_.

The band alignment has been obtained using (a) the independent units and (b) the surface terminated junction models, Figure 6. In both cases a Type‐II heterojunction is predicted, and the band offsets differ by about 0.1 eV only. Furthermore, the calculated offsets are fully consistent with the experimental ones.^[71]^ This example shows that the use of independent units, without explicit description of the interface, can be used in selected cases to predict the band alignment. However, as shown below, this does not hold true in presence of relevant polarization or charge transfer at the interface.

Finally, the impact of oxygen vacancies (O_v_) was also considered, as these are commonly present.[^99^, ^100^] O_v_ on TiO_2_ or in the contact region slightly decrease the offsets by 0.0–0.1 eV, while O_v_ on the SrTiO_3_ side (less stable) lead to an increase of the offsets. In general, the presence of oxygen vacancies has only a moderate impact on the band alignment of the TiO_2_/SrTiO_3_ junction.

### 3.4 InP/TiO_2_

**Figure 7:**
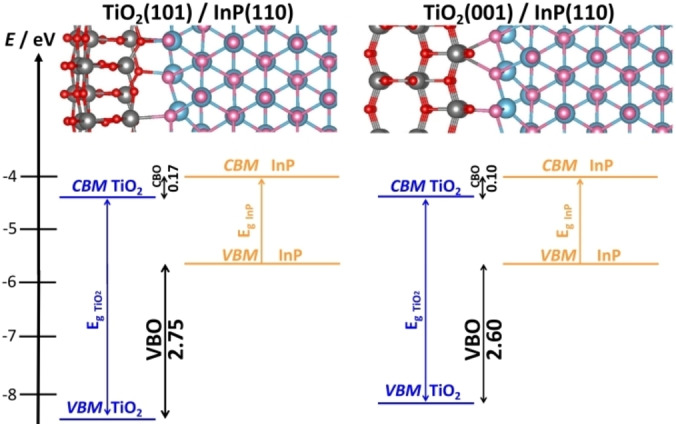
Band alignment of (a) InP/TiO_2_(101) and (b) InP/TiO_2_(001) heterojunction models. Grey: Ti; red: O; pink: In; light blue: P.

InP/TiO_2_ heterojunctions show high efficiency in the conversion of solar light, thanks to a strong visible light absorption by InP and an efficient migration of photogenerated electrons towards TiO_2_.[^29^, ^72^] The passivating TiO_2_ film also protects InP from water attack.^[31]^ We investigated the nature of InP/TiO_2_ by means of the VASP code and the HSE06 functional in order to unveil the role of the thickness of the TiO_2_ coating in the charge carriers migration.^[101]^ The InP/TiO_2_ interface can be created by combining InP(110) slabs with ultrathin TiO_2_ films of ∼0.6–0.8 nm thickness exposing either (101) or (001) surfaces. Here the use of the surface terminated junction approach to determine the band alignment is required due to the ultrathin nature of the titania layer. The formation of a InP/TiO_2_ junction is characterized by the formation of covalent In−O and Ti−P interface bonds, with the InP/TiO_2_(101) interface being more stable. Irrespective of the TiO_2_ surface in contact with InP, the system behaves as a Type‐II junction, where the TiO_2_ band edges are lower in energy than InP ones, Figure 7. A favorable Type‐II junction is retained for every thickness of the titania passivating film, but the highest efficiency is predicted for film thicknesses of 2–3 nm, as this provides a good compromise between favorable band alignment and efficient transfer of the photogenerated electrons to the surface.^[72]^ In this example, the role of the TiO_2_ surface used to create the junction is not crucial, as similar bands offsets are found for the two surfaces considered, (101) or (001), Figure 7.^[101]^ On the contrary, the TiO_2_ film thickness plays an important role in determining the band offsets and consequently the efficiency of the device.

### 3.5 WiO_3_/BiVO_4_

The third and last example of heterojunction made of two components is WO_3_/BiVO_4_. Photoanodes based on the WO_3_/BiVO_4_ heterojunction showed excellent solar‐to‐hydrogen conversion efficiency in water splitting.[^73^, ^102^, ^103^, ^104^] This is thanks to the visible light absorption of BiVO_4_ combined with the high electron conductivity of WO_3_. The nature of WO_3_/BiVO_4_ has been investigated with the aim to study the nature of charge transfer at the interface (CRYSTAL code, PBE0_DD_ and HSE06 functionals).^[105]^ Recent experimental results confirm that the (010) surface of BiVO_4_ is involved in the junction with WO_3_.^[106]^ A WO_3_ slab of 1.6 nm thickness exhibits converged properties while for BiVO_4_ 2 nm films are required to recover the bulk properties. The band alignment predicted by considering the separate units is qualitatively the same obtained simulating the explicit interface (surface terminated junction approach).

The role of charge polarization in this interface has been considered. This is an important aspect that should always been discussed, besides the position of the band edges. In fact, the band alignment determines the preferred direction of the photogenerated electrons and holes based on pure thermodynamic arguments; however, the creation of a large interface dipole due to the occurrence of a charge transfer may favor or disfavor charge carriers migration.[^5^, ^107^] In the case of WO_3_/BiVO_4_ the charge polarization at the interface is beneficial for charge separation since there is a charge accumulation on the BiVO_4_ side on the interface and a consequent charge depletion at the opposite side. This effect, together with the band alignment, explains the superior photoactivity of this heterojunction. A model limited to the separate units would not account for this phenomenon.

## 4 Type‐II Heterojunctions: Joining Different Facets of the Same Semiconductor

A Type‐II junction can be created also by joining differ surface terminations of the same semiconductor;[^57^, ^108^, ^109^, ^110^] in this case one generates a “surface junction”.^[3]^ Here we present two examples, one is the BiOIO_3_(010)/BiOIO_3_(100) interface, the second one involves the combination of two titania anatase facets, TiO_2_(001)/TiO_2_(101).

### 4.1 BiOIO_3_(010)/BiOIO_3_(100)

**Figure 8:**
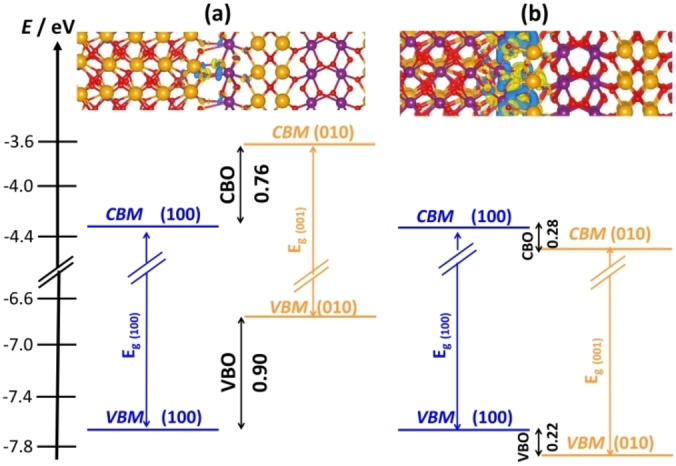
Band alignment in BiOIO_3_(100)/BiOIO_3_(010) (a) interface built from the most stable BiOIO_3_(100) and BiOIO_3_(010) surface terminations; (b) a thermodynamically more stable junction built from the most stable BiOIO_3_(100) surface and a less stable BiOIO_3_(010) termination. Only this latter model explains the observed band alignment of the BiOIO_3_(100)/BiOIO_3_(010) heterojunction.

BiOIO_3_ nanocrystals exposing (010) and (100) surfaces are excellent photocatalysts.^[110]^ However, if one considers the position of the VB and CB of the (010) and (100) surfaces of BiOIO_3_ one cannot explain the experimental trends. In fact, calculations done at the PBE0_DD_ level with the CRYSTAL code show unambiguously that the most stable (010) and (100) surfaces of BiOIO_3_ exhibit VBM and CBM positions which are reversed compared to experiment.^[111]^ To reconcile theory with experiment, it is necessary to invoke the formation of a junction between a less stable (010) surface termination of BiOIO_3_ with the most stable (100) one. When this is done new chemical bonds at the interface result in a thermodynamically stable system and a significant charge polarization at the interface. This junction not only provides the correct band alignment, but also exhibits a position of the energy levels that is fully consistent with the experiment, Figure 8.

This example shows that to rationalize the behavior of semiconductor heterojunctions it is important to describe the formation of explicit interfaces with atomistic precision, and to take into account the overall thermodynamic stability of the system, and not only the most stable surface terminations. This is a typical example where it is necessary to employ the “alternating slabs junction” or the “surface terminated junction” approaches.

### 4.2 TiO_2_(001)/TiO_2_(101)

The most common example of junction formed by interfacing two polymorphs of the same material is probably P25,^[112]^ a mixture of anatase (75 %) and rutile (25 %) widely used to benchmark the photocatalytic efficiency of new materials. An heterostructure formed by combining the (101) and (001) facets of anatase TiO_2_ provides interesting photocatalytic properties, even better than the reference P25,[^57^, ^108^, ^113^, ^114^, ^115^] and has been studied with the CRYSTAL code and PBE0[116] and PBE0_DD_ functionals. Two models have been used, the simple “independent units” approach and that based on “surface terminated junctions”. A two‐step process was followed in order to design a realistic model of the heterojunction. The superposition of (101) and (001) anatase facets leads to unacceptable strains and poor Ti−O match at the interface. Thus, a superlattice was created by rotating the two surfaces by an angle of ∼45°, so that the lattice strain is considerably reduced (6.3 % and 0.9 %) and a good cation‐anion match in the contact region is achieved.

**Figure 9:**
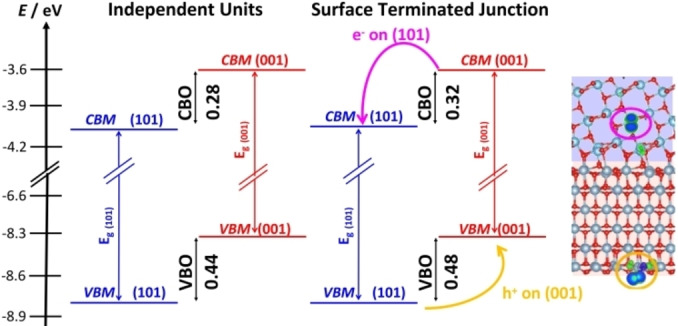
Band alignment of (001)‐(101) anatase surface junction (PBE0 results).

The resulting band alignment, obtained by considering electronic states on inner layers, is of Type‐II, Figure 9, and is predicted also by the independent units model.[^117^, ^118^] The picture provided by the band alignment was assessed by simulating the explicit formation of a hole in the VB and of an electron in the CB according to a singlet‐triplet excitation process.^[119]^ The vertical excitation was followed by a structural relaxation that leads to hole and electron localization and polaron formation.[^120^, ^121^, ^122^] The generation of charge carriers upon photon absorption starts at the (001) surface with formation of delocalized holes in the VB and electrons in the CB. The structural relaxation induces hole localization on a single O ion on the (001) side of the junction and migration of the excited electron to the (101) side, where it localizes to form a Ti^3+^ ion, as shown by the spin density plots, Figure 9. Of course, the process can be studied only when the interface is explicitly included in the model.

Then, we looked at the effect of Nitrogen doping on the properties of the TiO_2_(001)/TiO_2_(101) junction.^[123]^ While doping of oxide semiconductors has been addressed in several studies, much less attention has been dedicated to the combined effect of doping and formation of heterojunctions. The effect of N‐doping on anatase and rutile TiO_2_ has been studied extensively by some of us.[^124^, ^125^, ^126^, ^127^, ^128^] The presence of isolated N‐dopants results in localized defect states just above the VB of the material, thus increasing the absorption in the visible region of the spectrum. N‐dopants also favor the formation of O vacancies.

In a recent study, Sun et al. synthetized a junction formed by N‐doped (001)‐(101) anatase TiO_2_ surfaces.^[114]^ The new photocatalyst exhibits higher photoactivity under visible light with respect to the undoped samples, suggesting that the two main strategies to improve TiO_2_ performances (heterostructure and doping) can also work in a concerted way. A systematic analysis shows that both substitutional and interstitial N‐dopants localize preferentially at the interface. At higher dopant concentrations, also doping of the inner layers becomes energetically accessible.

The band alignment picture however is not strongly altered by the presence of the N‐impurities. This provides a solid theoretical basis for the experimental observation that the N‐dopants are not detrimental for photocatalysis, while they increase the visible‐light absorption capability. This is true also when the presence of the N‐dopants is combined with O vacancies, commonly present in real samples. The excess electrons associated to the O vacancy (Ti^3+^ 3d^1^) states are spontaneously transferred to the low‐lying N‐states, causing a remarkable decrease of the O vacancy formation energy. Despite these compensating mechanisms and internal charge transfers, the overall band alignment in N‐TiO_2_(001)/N‐TiO_2_(101) remains the same of the undoped junction.

## 5 Ternary Heterojunctions

In this section we discuss heterojunctions formed by three components, based on two examples. In the first one, TiO_2_/TiO_2_/ZnS, a Type‐II heterojunction is supposed to increase the efficiency due to a cascade process. In the second example, C_3_N_4_/SrTiO_3_/TiO_2_, the inclusion of a SrTiO_3_ buffer layer between C_3_N_4_ and TiO_2_ results in an inversion of the charge polarization at the interface and in a completely different behavior, from Type‐II in C_3_N_4_/TiO_2_ to Z‐scheme in C_3_N_4_/SrTiO_3_/TiO_2_.

### 5.1 TiO_2_/TiO_2_/ZnS

We have seen above that the anatase (101)‐(001) junction exhibits an efficient charge separation; the (101) side stabilizes the photogenerated electrons and the (001) side the photogenerated holes. An even better separation of charge carriers could be achieved adding a third component with higher hole‐stabilizing capability, such as a ZnS(110) layer grown on the anatase (001) surface. This results in a ternary TiO_2_/TiO_2_/ZnS composite, investigated with the CRYSTAL code and the PBE0 functional.^[117]^ New Zn−O and Ti−S bonds form at the interface between TiO_2_ and ZnS giving rise to non‐negligible structural reconstruction, also beyond the interfacial region.

The formation of a ternary compound has some effect on the band offsets. The computed band alignment is consistent with the experimental evidence,^[129]^ i. e. the ternary TiO_2_/TiO_2_/ZnS heterojunction promotes a charge carriers’ cascade. This has been confirmed by an explicit simulation of a photoexcited electron‐hole pair, which shows electron localization on the TiO_2_ side and hole formation on the ZnS side of the junction.

**Figure 10:**
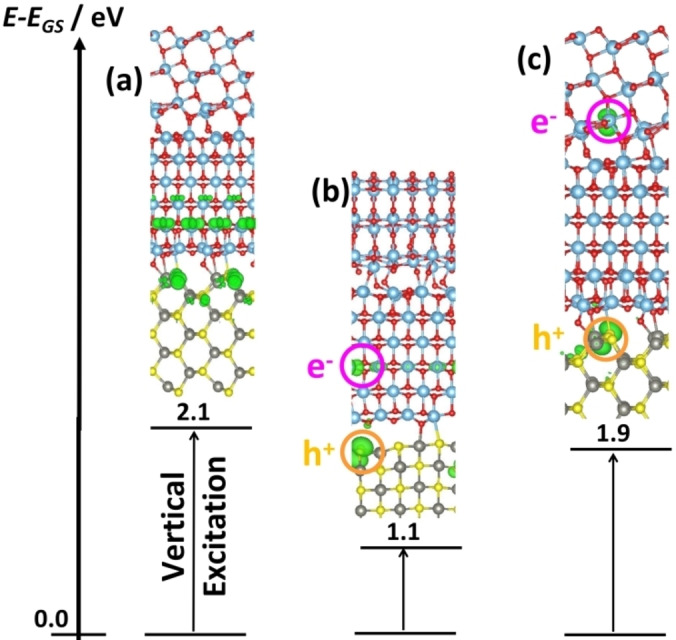
Triplet excited configurations in TiO_2_/TiO_2_/ZnS. (a) vertical excitation; (b) a relaxed configuration where h^+^ is localized on ZnS(110) and e^‐^ on TiO_2_(001) (two polarons). (c) a relaxed configuration where h^+^ is localized on ZnS(110) and e^‐^ in on TiO_2_(101). Spin density iso‐surfaces at 0.003 |e^‐^|/a_0_^3^ are shown in green. Light blue: Ti; red: O; yellow: S; grey: Zn.

However, the analysis of the band alignment alone is not sufficient to conclude on the direction and efficiency of charge carriers’ migration. The excitation of one electron from the VB to the CB (triplet state) results in a full localization of the electron on a Ti^3+^ ion of TiO_2_(001) and of the hole on a S site of the ZnS(110) surface, with formation of two polarons spatially separated by about 0.8 nm, Figure 10. The following step consists in localizing the electron on the TiO_2_(101) moiety, as predicted by the band alignment. However, this step implies a penalty of 0.8 eV due to Coulombic forces hindering the electron‐hole spatial separation. In this respect, the TiO_2_/TiO_2_/ZnS junction is predicted to have good photoactivity, but not to overperform the TiO_2_/TiO_2_ one. Coulomb forces act against the separation of charge carriers predicted based on the relative energy of the band edges. This highlights the necessity of explicitly including structural and electronic junction's effects, as well as charge carriers’ localization. Complex semiconductor architectures made by three or more units may exhibit effects that cannot be predicted by a simple inspection of the band edges of the system.

### 5.2 C_3_N_4_/SrTiO_3_/TiO_2_

**Figure 11:**
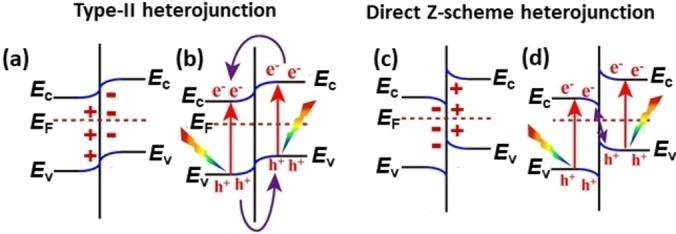
Type‐II and direct Z‐scheme heterojunction. (a) and (b) Type‐II photocatalyst: (a) Band bending at the interface due to the formation of the interface dipole; (b) Photo‐generated carriers migrate according to the alignment shown in (a). (c) and (d) Direct Z‐scheme photocatalyst; (c) Band bending at the interface due to the formation of the interface dipole; (d) Photo‐generated carriers migrate according to the alignment shown in (c). Reproduced with permission and adapted from Ref. [5]. Copyright: 2018, Elsevier.

The last case considered also shows that the simple inspection of the band alignment to predict the charge carriers separation may not be sufficient. This is the case of graphitic carbon nitride, g‐C_3_N_4_,[^130^, ^131^, ^132^, ^133^] a material which has attracted a lot of attention due to a high visible‐light activity, chemical stability, and facile synthesis. Beside these positive aspects, the activity of g‐C_3_N_4_ is deteriorated by a fast recombination of charge carriers,^[132]^ a problem that can be mitigated by creating a g‐C_3_N_4_/TiO_2_ (anatase) interface.[^134^, ^135^, ^136^, ^137^] Experimentally it has been shown that the TiO_2_ band edges are lower in energy than those of g‐C_3_N_4_;[^3^, ^5^, ^107^, ^135^] accordingly, g‐C_3_N_4_/TiO_2_ should behave as a Type‐II junction. On the contrary, photogenerated electrons and holes in g‐C_3_N_4_/TiO_2_ follow a different migration scheme, referred to as direct Z‐scheme.[^3^, ^5^, ^138^] Differently from a Type‐II junction where electrons and holes migrate towards the energetically most favorable component, Figures 11a and b, a different transport mechanism occurs in direct Z‐scheme photocatalysts. Here the photogenerated electrons on side A, with lower CB, recombine with the holes photogenerated on semiconductor B with a higher VB. In this way the photogenerated electrons in B and holes in A can be preserved, Figures 11c and d. Coherently with this scheme, in g‐C_3_N_4_/TiO_2_ electrons do not migrate from g‐C_3_N_4_ to TiO_2_, as expected for a Type‐II junction, and are retained on g‐C_3_N_4_. At the same time, holes do not migrate from TiO_2_ to g‐C_3_N_4_, but they tend to stay on the oxide. This change in behavior from Type‐II to direct Z‐scheme in g‐C_3_N_4_/TiO_2_ is due to an important effect: the generation of an interface dipole that determines a bend bending which prevents the flow of charge carriers as predicted by the Type‐II alignment, Figure 11.^[5]^

**Figure 12:**
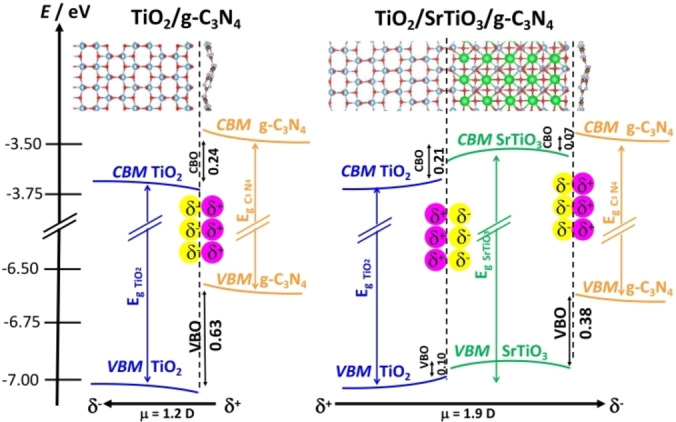
Band alignment and interface dipole in g‐C_3_N_4_/TiO_2_, a direct Z‐scheme photocatalyst, and g‐C_3_N_4_/SrTiO_3_/TiO_2_, a Type‐II heterojunctions. The change in behavior is due to the SrTiO_3_ buffer layer, and the inversion of the interface dipole.

The problem has been recently addressed by performing VASP DFT calculations with the HSE06 functional.^[139]^ The g‐C_3_N_4_/TiO_2_ interface was built by combining the g‐C_3_N_4_ and TiO_2_(001) units (as experimentally observed).^[140]^ In g‐C_3_N_4_/TiO_2_ the VB and CB bands of TiO_2_ are 0.63 eV and 0.24 eV, respectively, below those of g‐C_3_N_4_ (Type‐II alignment). However, the DFT calculations show the formation a large interface dipole of 1.2 D, with charge polarization δ^−^ on TiO_2_ and δ^+^ on g‐C_3_N_4_, which confer to the junction the typical direct Z‐scheme character, Figure 12. This provides a first principles rationalization of the observed behavior of g‐C_3_N_4_/TiO_2_ photocatalyst under irradiation and shows that the band alignment can be insufficient to predict the direction of charge carriers’ migration.

However, the direction of the charge carriers flow can be reversed by modifying the interface. This requires switching the direction of the interface dipole. A way to do this is to introduce a buffer layer between the g‐C_3_N_4_ and TiO_2_ components. In particular, the addition of a thin SrTiO_3_ layer, thus creating a ternary g‐C_3_N_4_/SrTiO_3_/TiO_2_ junction,^[141]^ leads to the desired result. The g‐C_3_N_4_/SrTiO_3_ interface is characterized by stronger covalent bonds compared to the g‐C_3_N_4_/TiO_2_ one. The band edges of SrTiO_3_ are between those of TiO_2_ and g‐C_3_N_4_, leading to a cascade sequence of Type‐II alignments, as for the TiO_2_/TiO_2_/ZnS case discussed above. The strong chemical bonds at the g‐C_3_N_4_/SrTiO_3_ side of the interface result in a large dipole of opposite sign compared to that of g‐C_3_N_4_/TiO_2_. The insertion of the SrTiO_3_ layer leads to the creation of an interface dipole that favors migration of photogenerated electrons toward TiO_2_ (δ^+^ charge polarization) and photogenerated holes toward g‐C_3_N_4_ (δ^−^ charge polarization). This picture is fully consistent with the experiments,^[141]^ thus providing a rationalization of the opposite behavior of g‐C_3_N_4_/TiO_2_, a direct Z‐scheme photocatalyst, and g‐C_3_N_4_/SrTiO_3_/TiO_2_, a classical Type‐II heterojunctions, Figure 12.

## 6 Conclusions

In this brief essay we have considered some examples of semiconductors heterojunctions as described by high‐level DFT calculations. The purpose of these studies is to define from first principles the nature of the band alignment, since only Type‐II junctions are favorable for the separation of photogenerated electrons and holes. Beside this, also the band offsets, the kind of surfaces involved, the direction and size of the interface dipole, etc. are essential for the rationalization of the behavior of existing photocatalysts, or for the design of new ones.

From a methodological point of view, the use of hybrid functionals or other self‐interaction corrected functionals is recommended, while less important is the choice of the hybrid functional used, provided that it guarantees a balanced description of the two (or more) components of the junction.

Different models can be adopted to determine the band alignment. In some cases, the use of the simple “independent units” model is sufficient to predict the band alignment. This occurs when no important charge transfer occurs at the interface. In the other cases, either the “alternating slabs junction” or the “surface terminated junction” models are employed, and the chemical nature of the interface is explicitly included. In both cases, the choice of the surfaces to be joined is crucial: this determines the amount of strain in the model, the thermodynamic stability of the interface, the occurrence of charge transfer, the final band offsets, etc. Both “alternating slabs junction” and “surface terminated junction” approaches make use of slabs to represent the bulk materials, and a proper study of the convergence of the electronic properties with slab thickness is required. The use of slabs, on the other hand, allows one to study quantum confinement effects when ultrathin layers of a semiconductor are deposited on another material.

Once the explicit model of the interface is defined, some other aspects need to be considered. One is the direction of the charge polarization at the interface. This results in an interface dipole that can facilitate the charge carriers’ separation, or act to modify the nature of the band alignment, for instance from Type‐II to a direct Z‐scheme. We have shown how in some systems the inclusion of buffer layers can be used to reverse the sign of the charge polarization and the nature of the band alignment.

Another aspect that is worth considering is the explicit formation of photogenerated electron/hole pairs by computing the triplet excited state of the system. The subsequent structural relaxation results in polarons formation and carriers localization on the two sides of the junction. However, this may result in a strong coulomb interaction that makes the charge carriers separation less favorable, despite the alignment of the bands.

From the few cases studied it does not seem that point defects or impurity atoms included in one or both sides of the junction have a major effect on the final alignment and band offsets. On the other hand, very few studies have been dedicated to this aspect, and further work is necessary to provide an answer to this question.

Finally, it must be mentioned that the presented models do not include solvent effects, that could become important.^[142]^ Future studies will be dedicated specifically on the modelling of solvent effects and the role played by the semiconductor/solvent interface on the band alignment.[^143^, ^144^]

## Conflict of interest

The authors declare no conflict of interest.

## Biographical Information

*Dr. Giovanni Di Liberto studied at University of Milan and graduated in 2015. He got a PhD in Chemistry developing theoretical methods for spectroscopy of complex systems, and now his research is devoted to the simulations of nanomaterials for solar light harvesting purposes. He has been visiting scientist at University of Barcelona in 2019. He obtained awards in 2018, and in 2020 from the physical‐chemistry and theoretical and computational chemistry divisions of the Italian Society of Chemistry*.

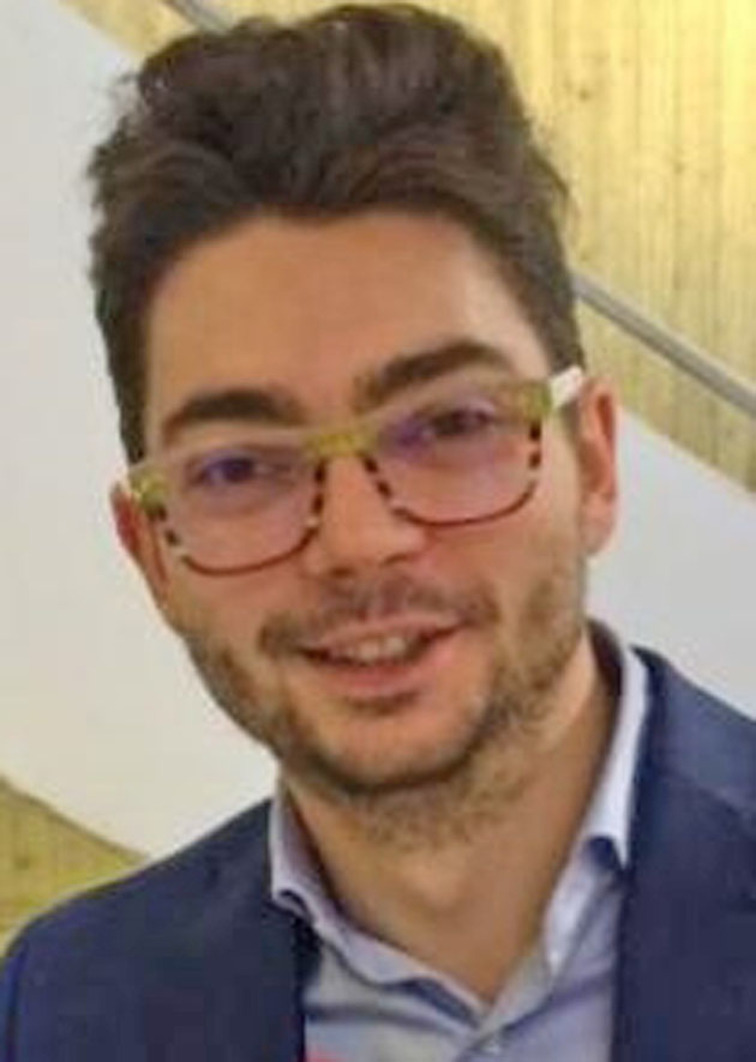

## Biographical Information

*Luis A. Cipriano graduated in chemical engineering in 2015 at the Universidad Autónoma Metropolitana ‐ Azcapotzalco. He is a PhD student in the Department of Material Science at the University of Milano Bicocca. His current research focus is on insulating and semiconductor oxides, supported metal on inorganic surfaces, and two‐dimensional oxides*.

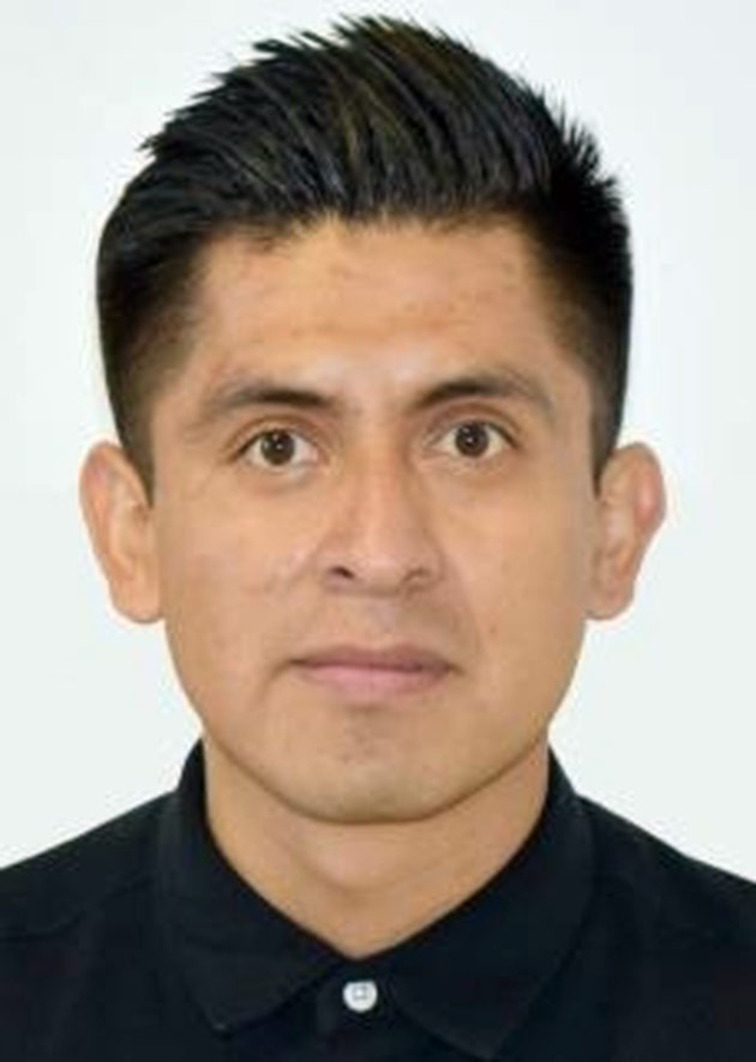

## Biographical Information

*Dr. Sergio Tosoni graduated in chemistry in 2003 from the University of Torino, where he also received his PhD in 2007. He worked as a post‐doc at the Humboldt University in Berlin (2008–2011) and at the University of Barcelona (2011–2013). He has been working since then at the Department of Materials Science at the University of Milano Bicocca, where he was appointed research associate in 2016 and as assistant professor in 2018. His research activity mainly concerns first‐principles simulations on the electronic structure and catalytic properties of oxide and oxide surfaces, as well as metal‐oxide interfaces*.

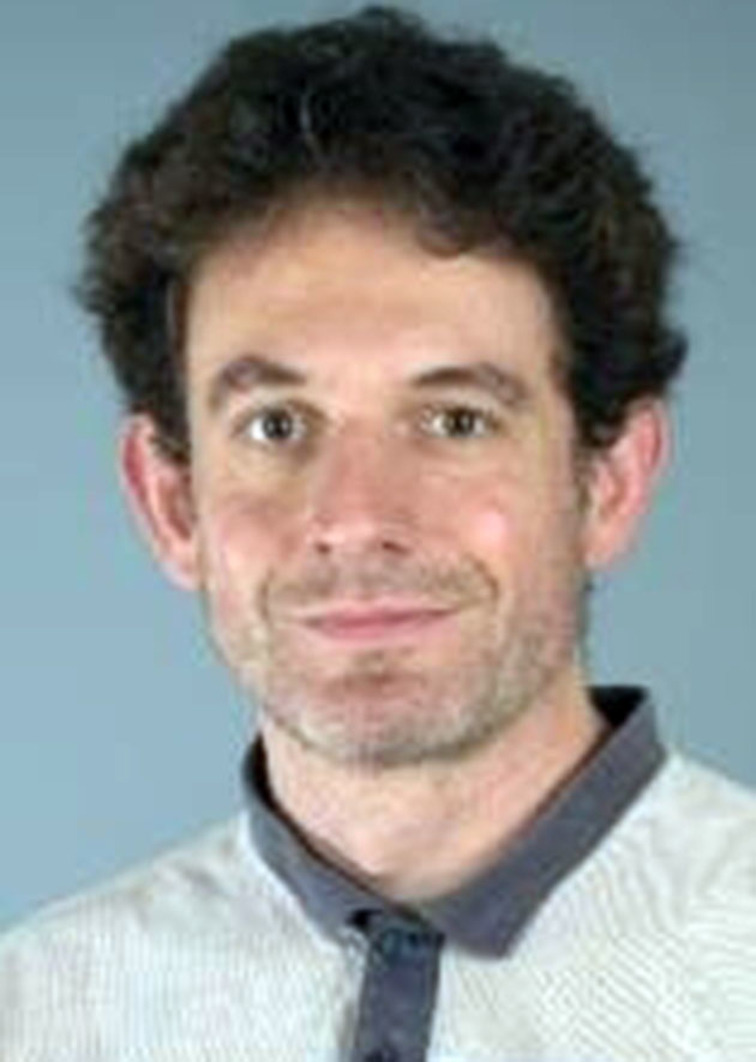

## Biographical Information

*Prof. Gianfranco Pacchioni received his PhD at the Freie Universität Berlin in 1984. He worked at the IBM Almaden Research Center, and at the Technical University of Munich. He is Full Professor at the University of Milano Bicocca where he has been Vice Rector for Research (2013‐2019) and Director of the Department of Materials Science (2003‐2009). He has published more than 500 papers (h index 88; WoS) and given nearly 500 invited talks on the electronic structure of materials for energy, catalysis and photocatalysis. He received several awards and is Fellow of the Accademia Nazionale dei Lincei (2014), the Academia Europaea (2012), and the European Academy of Sciences (2009)*.